# Clinical Study on the Melarsoprol-Related Encephalopathic Syndrome: Risk Factors and HLA Association

**DOI:** 10.3390/tropicalmed5010005

**Published:** 2020-01-01

**Authors:** Jorge Seixas, Jorge Atouguia, Teófilo Josenando, Gedeão Vatunga, Constantin Miaka Mia Bilenge, Pascal Lutumba, Christian Burri

**Affiliations:** 1 Institute of Hygiene and Tropical Medicine, NOVA University, 1349-008 Lisbon, Portugal; j.atouguia@gmail.com; 2 Global Health and Tropical Medicine R&D Center, NOVA University, 1349-008 Lisbon, Portugal; 3 Instituto de Combate e Controlo das Tripanossomíases, Luanda, Angola; josenandot@yahoo.com; 4 Hospital Geral de Luanda, Luanda, Angola; vmlgedeao@live.com; 5 Instituto Superior Politécnico Kalandula de Angola, Luanda, Angola; 6 Programme National de Lutte contre la Trypanosomiase Humaine Africaine, Kinshasa, Democratic Republic of the Congo; tshimbadi@yahoo.fr; 7 Tropical Medicine Department, University of Kinshasa, Kinshasa, Democratic Republic of the Congo; pascal_lutumba@yahoo.fr; 8 Swiss Tropical and Public Health Institute, 4002 Basel, Switzerland; christian.burri@swisstph.ch; 9 University of Basel, 4001 Basel, Switzerland

**Keywords:** human African trypanosomiasis, treatment, melarsoprol, adverse event, encephalopathy, human leukocyte antigen, association study

## Abstract

Melarsoprol administration for the treatment of late-stage human African trypanosomiasis (HAT) is associated with the development of an unpredictable and badly characterized encephalopathic syndrome (ES), probably of immune origin, that kills approximately 50% of those affected. We investigated the characteristics and clinical risk factors for ES, as well as the association between the Human Leukocyte Antigen (HLA) complex and the risk for ES in a case-control study. Late-stage Gambiense HAT patients treated with melarsoprol and developing ES (69 cases) were compared to patients not suffering from the syndrome (207 controls). Patients were enrolled in six HAT treatment centres in Angola and in the Democratic Republic of Congo. Standardized clinical data was obtained from all participants before melarsoprol was initiated. Class I (HLA-A, HLA-B, HLA-Cw) and II (HLA-DR) alleles were determined by PCR-SSOP methods in 62 ES cases and 189 controls. The principal ES pattern consisted in convulsions followed by a coma, whereas ES with exclusively mental changes was not observed. Oedema, bone pain, apathy, and a depressed humour were associated with a higher risk of ES, while abdominal pain, coma, respiratory distress, and a Babinski sign were associated with higher ES-associated mortality. Haplotype C*14/B*15 was associated with an elevated risk for ES (OR: 6.64; *p*-value: 0.008). Haplotypes A*23/C*14, A*23/B*15 and DR*07/B*58 also showed a weaker association with ES. This result supports the hypothesis that a genetically determined peculiar type of immune response confers susceptibility for ES.

## 1. Introduction

Human African trypanosomiasis (HAT, sleeping sickness) is a neglected tropical disease (NTD) caused by the protozoan parasites *Trypanosoma brucei gambiense* (Gambiense HAT, endemic in West and Central Africa) and *Trypanosoma brucei rhodesiense* (Rhodesiense HAT, endemic in Eastern and Southern Africa). The disease is transmitted through the bite of infected tsetse flies. HAT caused devastating epidemics at the beginning and the end of the 20th century [1]. As a consequence of intense and sustained control and surveillance activities which developed in endemic countries in the last 25 years, incidence levels were brought down to an all-time historical low (<1000) in 2018. However, HAT cases are still being reported in more than 20 countries in intertropical Africa. Most of the reported cases (98%) are of Gambiense HAT, which usually causes a chronic form of infection and in which humans are the main reservoir of the parasite. Rhodesiense HAT, a zoonotic disease that occasionally affects humans, usually causes a more acute form of disease and accounts for the remaining number of cases. HAT has been targeted by WHO for sustainable elimination by 2030. The essential component of the elimination strategy is the treatment of all diagnosed cases in order to deplete the parasite’s reservoir [2,3].

For over 50 years, successful treatment of sleeping sickness depended on correctly evaluating the degree of progression and severity of the disease. Following inoculation, infection remains temporally limited to the hemolymphatic system (hemolymphatic or early stage), but eventually progresses to central nervous system (CNS) invasion (meningo-encephalitic or late-stage). The transition between the early and late stage is insidious, and similar clinical features can exist in both stages [4]. The different penetration into the CNS, the pattern of adverse drug reactions, and the ease of use dictated the selection of the available drugs according to the disease stage until very recently. The early stage was treated with pentamidine or suramin because of the acceptable safety profile but insufficient drug levels in the cerebrospinal fluid (CSF). Late-stage *T.b. gambiense* HAT was typically treated with melarsoprol, and exceptionally with eflornithine. In 2009, the nifurtimox-eflornithine combination therapy (NECT) was added to the WHO essential drug list, replacing the more toxic drug, melarsoprol, as the first line of choice. NECT is, however, unreliable for the treatment of late-stage Rhodesiense HAT, and in this condition, melarsoprol remains the first choice [5]. In 2018, the European Medicines Agency gave a positive scientific opinion on the new oral monotherapy fexinidazole for use against both stages of Gambiense HAT, and subsequently, a marketing authorization was issued in the Democratic Republic of Congo [6]. The WHO updated their treatment guidelines accordingly in August 2019; melarsoprol is now limited to treatment of late-stage *T.b. rhodesiense* and as the last treatment option for relapse cases in *T.b. gambiense* HAT [7]. Clinical research to assess the efficacy of fexinidazole against *T.b. rhodesiense* has recently been initiated (ClinicalTrials.gov Identifier: NCT03974178).

Melarsoprol (Arsobal^TM^), introduced in 1949, was the first drug to efficiently treat both forms of late-stage HAT. Melarsoprol is the combination of melarsen oxide (a trivalent organic arsenical) with dimercaprol (British anti-lewisite, a heavy metal chelator developed as an antidote to arsenical-based nerve gases) [8]. Melarsoprol administration is associated with many adverse drug reactions, but the most severe and feared one is the encephalopathic syndrome (ES). ES is a badly defined, life-threatening neurological complication. ES may occur at any time-point after the first melarsoprol administration and up to 30 days after the last one, with a median and peak occurrence around day nine of treatment [9]. The incidence of ES ranges from 2 to 10% of all melarsoprol treatments, and is associated with a case fatality rate (CFR) of about 50% [10]. In a systematic review on ES, the average frequency appeared to be higher in Rhodesiense HAT (8.0%) than in Gambiense HAT (4.7%), with a CFR of 57% and 44%, respectively [11]. Despite this significant impact and more than 50 years of extensive melarsoprol usage, sound scientific knowledge regarding ES is still scarce.

Melarsoprol-associated ES is mostly described as consisting in the unpredictable and abrupt development of convulsions and intense agitation, followed by coma. However, descriptions of ES characterized only by convulsions or by the sudden onset of coma without previous convulsions also exist [12,13,14,15]. Based on restricted clinical–neuropathological correlation data, three independent forms of ES have been suggested, consisting in the separate existence of convulsions, coma, or mental (psychotic) changes. The severe and usually fatal coma type of ES is described as an acute hemorrhagic leukoencephalitis that includes severe abnormalities in the brain stem, with fibrinoid necrosis of small vessels and ring and ball haemorrhages. It is not clear whether these forms correspond to the same etiology or if they are different phenomena. The prognosis of the three hypothetical forms also varies: coma, and to a lesser extent, convulsions are associated with high mortality, whereas the mental form is benign and self-limiting [16]. Furthermore, convulsions, episodes of decreased level of consciousness, and psychotic manifestations are common displays of the severe CNS damage that occurs in late-stage HAT [17].

The cause of ES was generally assumed to be an immune phenomenon occurring mainly in the CNS and involving a complex host–drug–parasite interaction [18,19,20,21]. The Human Leukocyte Antigen (HLA) complex is a gene complex encoding the major histocompatibility complex (MHC) proteins in humans [22]. HLA is accepted as the major determinant of the immune response in humans. HLA determines the differentiation between “self” and “non-self”, and is involved in auto-immune diseases, as well as in determining susceptibility and resistance to several infectious diseases [23,24].

In order to improve the definition of the syndrome, to identify and quantify clinical risk factors for ES and for the ES case fatality rate, and also to determine whether an association between ES and Class I and II HLA antigens exists, we designed and conducted a case-control study with prospective data collection on the encephalopathic syndrome during treatment of late-stage Gambiense HAT with melarsoprol.

## 2. Materials and Methods

### 2.1. Study Design and Conduct

This was a case-control, non-blinded, non-randomized, non-interventive, multi-centre study, with prospective (i.e., before the patients were treated with melarsoprol) clinical data collection. Late-stage Gambiense HAT patients treated with melarsoprol and who developed or did not develop an encephalopathic syndrome were compared. The design of the study was nonparametric and aimed at testing whether or not the alleles of the HLA markers were distributed at random in patients suffering from ES [25]. The study was conducted from June 2002 in Angola and August 2002 in the DRC to November 2003.

Clearances were obtained from the ethical independent ethics committees of both Cantons of Basel (Switzerland) (Protocol approved under number 189/01), and of the Ministries of Health in Luanda (Angola) and Kinshasa (DRC). Informed consent was obtained from all participants or their legal tutors.

### 2.2. Study Sites

Participants were enrolled in six specialized HAT treatment centres supervised by the Instituto de Combate e Controlo das Tripanossomíases (ICCT) in Angola or by the Programme National de Lutte contre la Trypanosomiase Humaine Africaine (PNLTHA) in the Democratic Republic of Congo. In Angola, these centres were the Centro de Referência e Investigação de Viana (Luanda), N’Dalatando (Kwanza Norte Province), and Uíge (Uíge Province). In the DRC, participating centres were Mbuji Mayi (Kasai Oriental Province), Maluku (Kinshasa), and the Centre Neuro-Psycho-Pathologique (CNPP), Kinshasa.

### 2.3. Study Population

Inclusion criteria for cases and controls were as follows: (1) the patient had *T. b. gambiense* infection (confirmed either serologically or parasitologically in blood or lymph) and trypanosomes in CSF and/or CSF WBC count > 5 cells/mm^3^ and/or clearly defined neurological signs; (2) the patient was treated with melarsoprol; (3) the patient was conscious at admission (Coma Score ≥ 9 in the modified Glasgow Coma Scale) and was not convulsing before the start of melarsoprol treatment; and (4) mental changes of the psychotic type were not reported in the anamnesis or observed before the start of melarsoprol treatment. A patient was considered a case of encephalopathic syndrome when the following criteria were fulfilled: (1) onset of coma; (2) onset of convulsions; and (3) onset of severe mental symptoms, such as psychotic reactions and/or abnormal behaviour (aggressivity, severe confusion or disorientation) requiring a therapeutic intervention.

Exclusion criteria for both cases and controls were as follows: (1) the patient received melarsoprol in association with any other anti-trypanosomal drug; (2) patient follow-up could not be assured for 30 days after discharge.

For each ES case, three controls were enrolled. The controls were selected as the next three HAT patients following an ES case who underwent final discharge examination after having successfully completed treatment with melarsoprol, and did not develop ES during the follow-up period (to cover for the rare possibility of a late-onset ES).

### 2.4. Study Methodology

Detailed clinical data was obtained from every admitted late-stage HAT patient (and/or from relatives) before melarsoprol was initiated and recorded in a Case Report Form (CRF). When a patient developed an ES, the characteristics of the clinical presentation, of the management and of the final outcome of the syndrome were additionally inserted into the CRF.

Management of HAT patients (i.e., admission, diagnosis, treatment, discharge, and follow-up) and of ES cases in the participating centres followed the guidelines established by the respective national program. A microscopic test for *Plasmodium* was performed if the development of an ES was suspected in a patient. The test result was made available to the clinical staff for adequate management of the patient. No additional changes in routine procedures were introduced.

Blood sample collection for HLA typing was performed once the diagnosis of ES was established (cases) and at discharge for controls. Blood was collected onto filter paper cards (Generation^®^ Sample Collection Cards, Gentra Systems, MN, USA). Dried blood-spotted cards were inserted into sealable plastic bags and stored at room temperature.

### 2.5. Data Management

Data on CRFs was checked for inclusion and exclusion criteria and for completeness and consistency. Missing data and clarifications were obtained from the local investigators. Data from the CRFs was double-entered into two EpiData (http://epidata.dk/) databases—one for the demographic and clinical information of cases and controls, and the other for the description of ES cases. For analysis, the databases were merged and exported to the SPSS software package (SPSS for Windows, Rel. 15.5.0. 2002. SPSS Inc., Chicago, IL, USA).

### 2.6. HLA Typization

HLA typing was performed at the Molecular Genetics Laboratory of the Centro de Histocompatibilidade do Norte (North Histocompatibility Centre, CHN), Porto, Portugal, the reference facility for HLA typing for transplantation patients in Northern Portugal. Samples were typed for Class I HLA-A, HLA-B, and HLA-Cw molecules, and for Class II molecules of the HLA-DRB1 category using two different commercially available PCR-based reverse line blot assays that detect specific target DNA sequences by means of multiple immobilized sequence-specific oligonucleotide (SSO) probes [26,27,28,29]. The Dynal RELI™ SSO HLA Test (developed by Roche Molecular Systems, Inc. and manufactured by Dynal Biotech Ltd., Wirral, UK) for Class I molecules and the INNO-LiPA HLA-DRB1 Amplification and Hybridization Test (Innogenetics, Ghent, Belgium) for Class II molecules were used.

Hybridization was performed using an AutoLIPA (Innogenetics, Ghent, Belgium) automated assay processor, programmed to fit the Dynal RELI™ SSO HLA or the INNO-LiPA HLA-DRB1 hybridisation tests. To obtain the final HLA type, patterns were interpreted using the Dynal RELI SSO Pattern Matching Program, version 5.11 for Class I molecules (http://imgt.cines.fr) (Lefranc, 2003) or the LiPA interpretation software LiRAS™ for Class II molecules (using the latest available version from Abbott).

### 2.7. Statistical Analysis

Clinical data of ES cases and controls were compared using Pearson’s Χ^2^ or Fisher’s test. Risk factors for ES and death from ES were assessed by calculating the corresponding Odds Ratio (OR) and the 95% confidence interval (CI) for binomial variables. The numeric variables’ age and white blood cell count (WBC) in CSF were split into quartiles, and the Mann–Whitney test was used to compare medians; standard residual values while applying the Χ^2^ test were checked for values above > [1.96]. For evaluation of the body mass index (BMI) and patient general status on admission, proportions were compared by cross tabulation. Three categories were defined for BMI, corresponding to severe malnutrition (BMI ≤ 18), moderate malnutrition (BMI > 18 and ≤20), and normal nutritional status (BMI > 20). General status was categorized as “Bad”, “Fair”, or “Good”.

For the analysis of the relationship between HLA type and the frequency of ES, allele frequencies were compared among cases and controls by determining the *p* value of the Χ^2^ test applied to a 2 × 2 contingency table. OR and CI were obtained for alleles showing a significant *p* value. Haplotype frequencies were calculated assuming that the two allele loci were in linkage disequilibrium (LD). LD parameter ∆ was obtained by constructing a 2 × 2 contingency table and applying the formula originally described by Bodmer and Bodmer in 1970 [30]. LD ∆ was then added to the product of allele frequencies. Significant differences in haplotype frequency in cases and controls were subsequently obtained using the Χ^2^ test with Fisher’s exact test, with the OR and CI.

## 3. Results

Case Report Forms of 76 potential cases of encephalopathic syndrome (ES) were received. After checking for inclusion and exclusion criteria, 69 ES cases and 207 control patients were retained and included for analysis. The center of enrolment and outcome of ES patients are described in Table 1.

Thirty (43.3%) patients were female and 39 (56.5%) were male. The age of ES patients ranged from 4 to 67 years, with median and mean values of 26 and 30.6 years, respectively (std. deviation: 14.6 years). Three patients were less than 10 years old, and two patients were above 60 years. Ethnic heterogeneity in the study population was considerable. In Angola, patients belonged to the Kimbondo, Kikongo, Ovimbundo, and Bakongo ethnic groups. The determination of ethnic groups was not available in the DRC, since only the village of birth of the patient was recorded, due to difficulties found in attributing the correct ethnic group to individuals. All but five patients had parasitologically confirmed HAT. These five patients had been previously treated with pentamidine; late-stage HAT diagnosis was established according to a positive card agglutination test for trypanosomiasis (CATT) and more than 5 WBC/mm^3^ in CSF. All patients were treated according to the abridged IMPAMEL schedule (2.2 mg/kg/day of melarsoprol for 10 consecutive days).

### 3.1. Characteristics of the Encephalopathic Syndrome

#### 3.1.1. Clinical Features

ES was mainly characterized by convulsions followed by coma. Variations consisting of isolated convulsions or coma without convulsions occurred with lower frequencies (Table 2). Convulsions were observed in 85.5% of all cases, and 71% of the convulsive episodes were multiple. Convulsions led to coma in 55% of all ES cases, whereas coma not preceded by convulsions was observed in 14.5% of all ES cases. For coma characterization, we used a modified Glasgow Score, where unarousable coma is associated with a score of 7 or less. During the ES episode, the mean minimal score was 4.8, with a median score of 5 (Std. Deviation: 2.6) and the mean maximal score was 6.3, with a median score of 6 (Std. Deviation: 2.5). Additional signs, such as fever and maculopapular cutaneous lesions, were more frequent in the subgroup developing convulsions followed by coma (52% of cases developing fever and maculopapular lesions) than in the coma (20%) and convulsive (5%) sub-groups, respectively. The mental type of ES was not observed in the study.

Additional symptoms and signs characterizing ES cases and corresponding degrees are shown in Table 3. Degree 1 was defined as transient, mild, or localized. Degree 2 was defined as durable and needing intervention, intolerable, severe, or diffuse. For fever, Degree 1 was defined as 37.5–38.9 °C, and Degree 2 as more than 39 °C. For tachycardia, Degree 1 was defined as less than 100 bpm, and Degree 2 as more than 100 bpm. For hypotension, Degree 1 was defined as systolic blood pressure less than 80 mmHg, and Degree 2 as shock. Fundoscopic examination was not performed in ES patients.

More than half of the ES patients developed a deep malaise, entered a confusional status, and had fever. Apathy and agitation periods were frequent and could alternate. Two thirds of the patients were tachycardic, but hypotension was present in only 16% of them. Respiratory distress, consisting mainly in irregular respiratory patterns, frequently complicated the advanced phase of ES, preceding death. Nausea and vomiting were present in less than one quarter of the patients. A maculopapular eruption, conjunctival hyperaemia (red eye syndrome), or facial oedema were observed in half, one third, and one fifth of the patients, respectively. Additional signs observed were vertigo in up to one third of the patients and psychotic manifestations (hallucinations, delirium, panic attack, or aggressive behaviour), present in around one tenth of the patients. Damage to the cortical spinal tract, demonstrated by the Babinski sign, was present in one third of the patients. Meningeal irritation, demonstrated by neck rigidity, was observed in only 10% of the patients. Clinical signs of bleeding disorder were not observed.

#### 3.1.2. Laboratory Evaluation of ES

Laboratory evaluation (haemoglobin, leukocyte count in blood and in CSF) within the duration of the ES episode was performed in 15 patients. Neither the leukocyte differential blood count nor the platelet counts were available. ES patients did not show severe anaemia or significant changes in the leukocyte blood count. To check for bacterial meningitis, a Gram-stained smear was performed in 14 CSF samples, always with a negative result. In 11 patients, CSF glucose content was also obtained, but was not simultaneously measured in blood. Half of the patients had glucose levels in CSF close to or below the accepted inferior limit of 50 mg/dL. No increase above the WBC count in CSF on admission was observed during ES. *Plasmodium* smear was negative in all ES cases evaluated for CSF parameters. Additional laboratory parameters for hepatic, renal, respiratory, and metabolic evaluation were not available at the Centres.

#### 3.1.3. Time Point and Outcome for ES

The mean and median numbers of melarsoprol applications associated with the development of ES were 8.5 and 9 doses, respectively (Std. Deviation: 1.9; range: 2 to 10 doses). Melarsoprol was administered on consecutive days in all cases, except in one hypertensive patient due to hypertension peaks, in whom with treatment was interrupted for 2 days; 47.8% of patients developed ES with the 10th dose of melarsoprol. The number of melarsoprol administrations preceding the development of ES was not significantly different whether the initial manifestation of ES consisted of coma or convulsions (*p* value of the Mann–Whitney test: 0.26; all observed counts not statistically different from to those expected: all std. residuals below 1.96).

**Figure 1:**
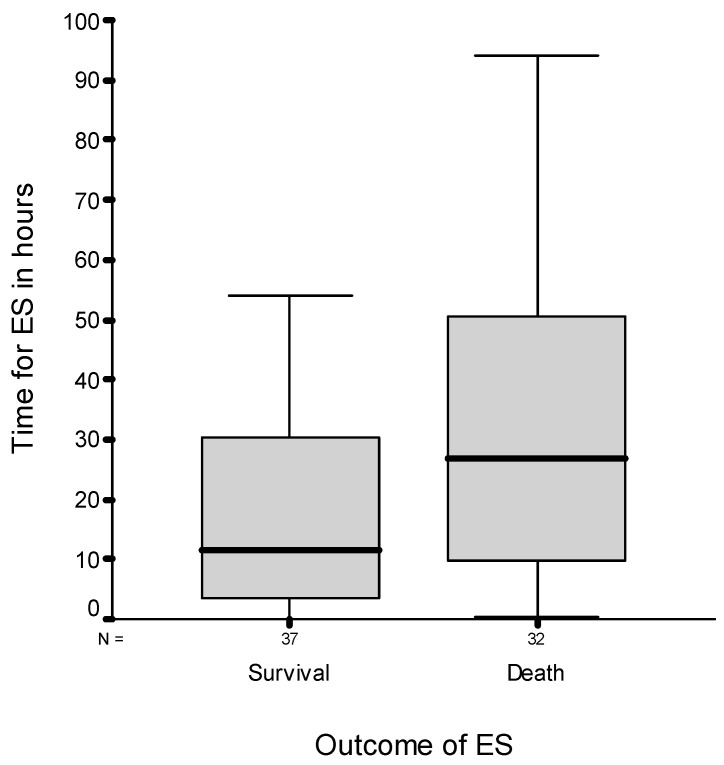
Time interval in hours for the occurrence of ES after the last melarsoprol application according to the final outcome. The inbox line in bold indicates the median. Outliers and extremes are not shown.

The mean time interval between the last application of melarsoprol and start of ES was 35.4 h. This interval is the time between melarsoprol administration and the development of symptoms and signs of an impending encephalopathy, during which subsequent doses of the drug are withheld. Half of the patients developed ES within 23.5 h after the last application, and 75% did so 47.7 h within the last drug application. Figure 1 shows the median time interval distribution for the development of ES after the last melarsoprol application, discriminated according to the outcome of ES. The interval for the occurrence of ES after the last dose of melarsoprol before ES was not significantly different between survivors and fatalities (Mann–Whitney test, *p* value: 0.08).

Encephalopathic syndromes led to a fatal outcome in 32 patients (46.4%). On average, death occurred 2.5 days after diagnosis of the syndrome (median: 1 day; std. deviation: 2.9 days; range 0–13 days). 80.6% of the patients died within three days after developing ES.

On average, ES manifestations ceased 2.2 days after initiation of the episode in the 37 surviving patients. The majority of surviving patients (73%) did not show any sequelae on discharge. When present, sequelae consisted in tremors (three cases), mental changes (confusion, euphoria, aggressivity) (three cases), paraplegia (two cases, one with urinary retention), speech disturbances (polylalia, dysarthria, aphasia) (three cases), and walking disability (one case).

#### 3.1.4. Patient Management

All centres used corticosteroids (prednisone, hydrocortisone, occasionally combined with promethazine), antimalarials (chloroquine, sulfadoxine-pyrimethamine, or quinine), and anthelminthics (mebendazole, levamisole) and multivitamins for “patient preparation” before melarsoprol administration, aiming at reducing its toxicity.

All ES patients were managed with corticosteroids, except two patients (that survived). Half of the patients under corticosteroid therapy for ES died. Convulsions were managed with standard doses of diazepam and/or phenobarbital, which were frequently used simultaneously with promethazine. The difference in outcome in patients using the three diverse types of antipyretic (paracetamol, acetyl salicylic acid, or dipyrone) was not significant. Antibiotics (mainly ampicillin, in one patient in combination with gentamicin) were used when a severe bacterial infection, usually respiratory, was suspected during ES. The use of quinine, mannitol, furosemide, and adrenaline was associated with high mortality. No evident anomalies in the prescribed dosage of the drugs were detected.

Intravenous fluids (5% glucose in water or Ringer lactate) were used in 61 patients. A urethral catheter was inserted in 40 patients, and a nasogastric tube was used in 13. However, monitoring of fluid and caloric input/output was never recorded. Medical oxygen was not available in the centres.

### 3.2. Risk Factors for the Encephalopathic Syndrome

#### 3.2.1. Clinical Factors

All variables in the questionnaire were analysed to determine if they constituted risk factors for the development of ES or for death from ES. Table 4 shows the variables obtained on admission that yielded a significant *p* value (<0.05) highlighted in bold. Selected variables with a higher *p* value when the OR indicated a tendency (≥2), even when the lower limit of the 95% CI was below 1, are also indicated. All other variables were below this threshold, and yielded insignificant *p* or OR values.

Patients reporting oedema in history or with apathy and/or depressed humour in the physical exam had a significant risk of developing ES. Patients who reported with abdominal pain, conjunctivitis, or pruritus in the anamnesis had a higher risk of dying from ES.

During the ES episode, coma (OR: 2.4, *p* value < 0.001), respiratory distress (OR: 5, *p* value: 0.002) and a Babinski sign (OR: 3.8, *p* value: 0.04) were significantly associated with death. Coma usually developed after convulsions, and respiratory distress often appeared as a terminal event. Respiratory distress was apparently not due to iatrogenic pulmonary oedema (no reference to physical signs of it was found). Tachycardia, hypotension, and, to a lesser extent, agitation were also marginally associated with death.

#### 3.2.2. Laboratory Data

Laboratory evaluation on admission showed that none of the measured biological markers (Haemoglobin, WBC count in blood or in CSF) was clearly associated with the occurrence of ES. CSF WBC count on admission was analysed by dividing the results of the count in quartiles. The limit for the first quartile was found to be 100 WBC/mm^3^ in the CSF. Additionally, the more conventional division of WBC count in CSF (less than 20 cells, 21 to 100 cells, and more than 100 cells in CSF) was also used in analysis (data not shown). Trypanosomes were found more frequently in the lymph (*p* value: 0.2, OR: 1.8) and in the CSF (*p* value: 0.2, OR: 2.2) of patients who developed ES, but possibly due to the small numbers, the difference was not statistically significant.

#### 3.2.3. Concomitant Diseases

Of the 58 patients tested for malaria during ES, 13 (22.4%) were positive (thick smear) and 8 (61%) died, while 45 (77.6%) were negative and 23 (51%) died.

Diagnosis of other concomitant diseases on admission included oral candidosis, lymphatic filariasis, pulmonary tuberculosis, and orchitis in one patient each. The patient with oral candidosis did not develop ES, and the three others died from ES. During ES, a clinical diagnosis of respiratory infection was clearly established in two patients, and thereof, one died.

#### 3.2.4. HLA Type

Alleles for every tested HLA category could be clearly determined for 62 ES cases and 189 controls. Samples showing any ambiguity in allele determination were excluded from the analysis. The statistical association between the HLA type and the development of ES is shown in Table 5.

Deducted haplotype C*14/B*15 was associated with a risk more than 6.5 times higher of developing ES. The association is significant (*p* value: 0.008), but the upper limit of the CI is considerably large. Haplotype A*23/C*14 was also found to be potentially associated with ES (*p* value less than 0.05), with a risk nearly 10 times greater of developing ES with this haplotype, but the inferior limit of the CI is below one, suggesting that a greater number of patients is needed for confirmation. A*23/B*15 and DR*07/B*58 showed a tendency for association with ES, but the level of significance for these allele combinations is above a *p* value of 0.05.

## 4. Discussion

### 4.1. Characteristics of ES

Until today, this is the largest single study on ES patients that has ever been conducted. So far, the results were only published as a chapter of a PhD thesis [11]. When the study was carried out, melarsoprol was still the only routine option to treat late-stage HAT. Still, the findings have relevance today, since *T.b. rhodesiense* is still treated with this organoarsenic drug, even if we cannot affirm with complete confidence that the risk factors we found in our study will apply to ES associated with this form of sleeping sickness. Nevertheless, our findings are important because they contribute to the understanding of immunological processes in the development of severe adverse drug reactions in the brain.

The results are in line with the majority of publications describing ES. However, they do not support the hypothesis formulated in two publications that three separate forms of the phenomenon exist [9,16], which may rather be the result of observational bias. Our findings, derived from a multi-center prospective study specifically designed to investigate ES and therefore being potentially less bias-prone, suggest that full-blown ES consists in multiple convulsions, where half of the cases are followed by a deep coma. Variations consisting in isolated convulsions or coma without convulsions occurred with lower frequencies around this main pattern. Additional signs, such as fever and maculopapular cutaneous lesions, were more frequent in the subgroup developing convulsions followed by coma than in the coma and convulsive sub-groups. Patients showed profound malaise, mental confusion, and apathy alternating with agitation and tachycardia. Additional signs consisting in conjunctival hyperaemia and facial oedema were present in 1/3 and 1/5 of the patients, respectively.

The exclusively mental type of ES was not observed in the study. Four cases were initially enrolled as belonging to the exclusively mental type of ES. They were, however, excluded from analysis after the presence of severe mental changes in anamnesis in the course of HAT was detected prior to melarsoprol application. This suggests that previous descriptions of this hypothetical type of ES may also suffer from an observational bias, with mental changes already present but not detected in the patient history or under-evaluated in clinical examination. Alternatively, we may speculate that if an exclusively mental type of ES exists, the frequency of the phenomenon is probably very low.

Our patients were all treated with the consecutive 10 day melarsoprol schedule, which has been recommended by the International Scientific Council for Trypanosomiasis Research and Control for late-stage Gambiense HAT in 2003 and by the WHO in 2013 [5], in substitution of older complicated schemes [31]. In accordance with previous data [9], we could confirm that ES occurs preferably after the eighth dose of melarsoprol, and in half of the cases, with the 10th dose. The time-point for occurrence and the prognosis of ES have been the basis for discussions about the etiology and severity of the phenomenon. It has been shown that ES occurs at the same average point in time, independent of whether the abridged 10 consecutive days’ constant dosage melarsoprol scheme or an older scheme of progressively increasing intermittent dosage (three series of 4 days with a 7 day interval) is used [31]. A subsequent study showed that both the coma and the convulsive types of ES also tend to occur at the same time-point [9]). In our study, the number of days elapsing before ES developed was similar for both initial manifestations of ES (coma or convulsions) and for the outcome (survival or death). A higher lethality of the hypothetical coma type of ES is described in the literature [9,16]. Our findings indicate that the highest mortality (36.2%) was instead associated with convulsions followed by coma (observed in 55% of the ES cases). Together with our findings on HLA involvement, these results give evidence that ES is an immunological phenomenon and that the different clinical presentations correspond to different severities of the same pathogenetic mechanisms. Further evidence might come from clinical–neuropathological correlation studies, as they would permit confirming the similarities between the neuropathological characteristics of ES and acute hemorrhagic leukoencephalitis (AHLE, Hurst disease), which is considered the most severe form of acute disseminated encephalomyelitis (ADEM). Patients with Hurst disease usually develop a severe CNS condition but, like in ES, different clinical expressions exist [32]). The anatomopathological changes found in AHLE [33] are very similar to the ones described in the coma type of ES by Haller in 1986.

In the literature, the development or intensification of unspecific signs, such as fever, headache, nausea, vomiting, dizziness, tremors, and conjunctival hyperaemia are considered leading signs of ES [34]. The search for distinct heralding signs of ES was an objective of our study protocol, but except for fever, they were generally not reported. However, the fact that clinicians stopped melarsoprol administration in an average of 35.4 h before the inclusion criteria for ES were reached indicates that they suspected the development of the complication.

In 80% of the patients reaching a fatal outcome, death occurred within three days. Surviving patients mostly recovered completely from ES in less than three days. These facts stress the need for careful and adequate patient surveillance while under treatment with melarsoprol so that a prompt diagnosis of ES can be made, and adequate therapeutic measures swiftly initiated. A lethal outcome was the result of ES in nearly half (46.4%) of the cases, with non-significant differences among the participating centres, which is within the range of mortality for ES in Gambiense HAT found in the literature.

None of the limited laboratory variables (haemoglobin and leukocyte count in blood and in CSF) evaluated during ES (rarely reported in the literature) in 15 patients proved useful in the confirmation of an ES diagnosis. We may conclude that there are no useful laboratory markers of ES available for use in the resource-limited rural African health facilities. However, glucose levels in CSF were close to or below the accepted inferior limit of 50 mg/dL in half of the patients, suggesting that low glucose levels in CSF, a potential triggering factor for convulsions, may be frequent in ES. To the best of our knowledge, this original finding could be important for patient management. The CSF Gram stain obtained in 14 ES patients was always negative, showing (with some degree of confidence) that ES patients in the study did not suffer from a confounding and potentially treatable bacterial meningoencephalitis.

### 4.2. Risk Factors for ES

Haplotype C*14/B*15 was significantly associated with ES. Three additional haplotypes (A*23/B*15, A*23/C*14, and DR*07/B*58) showed lesser degrees of association with ES, but sample size was insufficient for adequate statistical power. Alternatively, we may consider that the genetic component of ES could be polygenic or associated with a single major locus with other loci of small effect. The ethnical diversity observed in the clinical study was considerable, and the low numbers in the different ethnic groups was not sufficient to allow for sufficiently statistically powered ethnic stratification. As usual in genetic association studies, confirmation of these results would be needed in other populations. However, our data does suggest that there is a genetic predisposition for ES. This finding is in line with the described association between acute hemorrhagic leukoencephalitis (AHLE, Hurst disease), and particular, HLA haplotypes [32]. Since the physical map of the HLA complex genes is now available, it would be possible to explore the molecular basis of ES. Linkage of the Class II allele found in the study with Class III genes or non-HLA genes involved in the immune response, especially those related to the synthesis of the cytokines and mediators described in late-stage HAT could be explored to gain further insight into the etiology and pathogenesis of ES.

The following risk factors were consistently identified for ES and for death from ES, respectively: oedema, apathy, and a depressed humour; as well as abdominal pain, pruritus, and conjunctivitis. Additional variables, such as diarrhoea in patient history and facial paralysis and a positive Babinski sign on examination yielded elevated OR values, but their *p* values were below statistical significance. However, the identified symptoms and signs may be transitory and elusive, and may need a careful and detailed evaluation of the patient, which clearly limits their practical value, especially in the African setting. The absence of a clear association between elevated WBC in CSF and presence of trypanosomes in body compartments conflicts with findings from previous studies [9,10,18].

Concomitant infections, particularly malaria, have long been suspected of increasing the risk of ES [9,35]. The issue of concomitant infections and melarsoprol adverse events, including ES, was addressed in a large cohort of patients treated with a concise 10 day schedule [36]. Malaria parasites were found in 22.7% (40/176) of the patients suffering from ES, despite the fact that, as in our study, all patients received a full course of chloroquine or sulfadoxine and pyrimethamine or quinine during the preparation period prior to melarsoprol. In our study, 22.4% (13/58) of the patients showed a positive smear during ES. The role of *Plasmodium* infection in ES is difficult to establish, and its analysis is inherently associated with the quality of the malaria diagnosis, which was frequently low in the participating centres.

Malnutrition is very frequent in patients suffering from HAT. In one report, more than 20% of adults and 56% of the children with Gambiense HAT were affected [37]. A deficient nutritional status and a bad general status are empirically considered as having a significant impact on the risk for ES. However, in our clinical study, neither the general status nor the BMI was correlated with ES or death from ES, which is in accordance with the findings from a large and adequately powered melarsoprol trial (IMPAMEL II) [35].

### 4.3. Patient Management

Because this was a non-interventive study, and given the multiplicity of the drugs used and the low number of patients enrolled in some centres, we could not evaluate the usefulness of the individual drugs (prednisone, antimalarials, anthelminthics, and multivitamins) used for prevention of ES. Corticosteroids are presently accepted as beneficial in the prevention of ES and widely used for this purpose, but the validity of this intervention is not based on solid evidence [10,11,34].

Management of ES patients in our study with dexamethasone was associated with lower mortality (38.5%) when compared to hydrocortisone (50%), which was the most frequently used corticosteroid. Hydrocortisone has a high sodium retention action when used at the dose needed to reach significant anti-cerebral oedema action, and may contribute to water retention and pulmonary oedema. Dexamethasone, used as in cerebral oedema, offers a more adequate pharmacological profile, and this could explain the tendency for a lower mortality observed with this drug in our study. Limited evidence shows that methylprednisolone, in high (5 to 10 mg/kg/day) or very high (1000 mg/day for an adult) doses, may be useful in critical patients with viral encephalitis, acute disseminated encephalomyelitis (ADEM), and AHLE [38,39]. Such high-dose methylprednisolone schemes could be suitable in ES, given the similarities between these conditions and ES.

Quinine, furosemide, mannitol, and adrenaline can promote or increase cardiovascular arrhythmias, hypoglycaemia, and fluid and electrolyte imbalance, which, in addition to the frequent severe clinical deterioration of ES patients, may explain the high mortality associated with these drugs in our study. Their use in a critically ill ES patient is hazardous (especially without good monitoring equipment and as a combination) and should probably be limited to patients with accurately diagnosed malaria, strong evidence of fluid overload, raised intracranial pressure, shock, or severe bacterial infection.

Human African trypanosomiasis is nowadays considered a rare disease targeted for elimination [40]. Melarsoprol use in HAT is presently limited to rescue therapy of cases refractory to other drug treatments in Gambiense HAT and to Rhodesiense HAT late-stage patients, meaning that melarsoprol-related ES will become a rare event. In our opinion, this also means that patients in need of melarsoprol treatment should preferably be referred to specialized centres where knowledge on the correct management of complications associated with the use of this powerful but dangerous drug persists.

## 5. Conclusions

In conclusion, our study obtained a more precise definition of ES, which is useful in the correct diagnosis of the condition. The observed clinical risks factors for ES were elusive. A significant statistical association between Class I haplotype C*14/B*15 and ES was found. Patients expressing this haplotype have an almost 6.5 times higher risk of developing ES. Additionally, three other haplotypes were found to be possibly related to ES. This finding corroborates our hypothesis of an immunological involvement in the pathogenesis of ES. This result indicates that a genetically determined individual susceptibility exists that produces a peculiar type of immune response in the central nervous system that, in combination with the presence of trypanosomes and melarsoprol, results in a catastrophic condition in many aspects similar to acute hemorrhagic leukoencephalitis (AHLE, Hurst disease).

## Figures and Tables

**Table 1:** Origin and outcome of enrolled ES patients.

HAT Treatment Centre	Number of ES Patients	Deaths
Mbuji Mayi (DRC)	24 (35%)	10 (41.6%)
Viana (Angola)	21 (30%)	10 (47.6%)
Maluku (DRC)	8 (12%)	4 (50%)
Uíge (Angola)	7 (10%)	4 (57.1%)
N’Dalatando (Angola)	6 (9%)	3 (50%)
CNPP (DRC)	3 (4%)	1 (33.3%)

**Table 2:** Type of manifestation of ES observed and discriminated according to the outcome. The existence of fever and/or maculopapular cutaneous eruption (urticaria) is also indicated.

Type of Manifestation of ES	Cases N/(%)	Outcome Survival n/%	Additional Signs n	Outcome Death N/%	Additional Signs n
Convulsion and coma	38/(55)	**13/(18.8)**	Fever in 3 Urticaria in 3 Fever and urticaria in 1	**25/(36.2)**	Fever in 15 Urticaria in 7 Fever and urticarial in 5
Convulsion without coma	21/(30.5)	**19/(27.5)**	Fever in 6 Urticaria in 4 Fever and urticaria in 2	**2/(2.9)**	Fever in 1
Coma without Convulsions	10/(14.5)	**5/(7.2)**	Fever in 2 Fever and urticaria in 1	**5/(7.2)**	Fever in 2 Urticaria in 1
Totals	69/(100)	**37/(53.6)**	Fever in 11 Urticaria in 7 Fever and urticaria in 4	**32/(46.4)**	Fever in 18 Urticaria in 8 Fever and urticaria in 6

**Table 3:** Symptoms and signs characterizing ES, according to the global frequency and discriminated for the degree of severity.

Characterization of ES Symptoms and Signs	Frequency (Global) % (n)	Frequency (Degree 1) %	Frequency (Degree 2) %
Malaise	79.7 (55)	20.3	59.4
Confusional state	78.3 (51)	26.1	52.2
Fever	69.6 (48)	52.2	17.4
Agitation	65.2 (45)	34.8	30.4
Tachycardia	60.9 (42)	39.1	21.7
Respiratory distress	59.4 (41)	36.2	23.2
Headache	56.5 (39)	37.7	18.8
Apathy	56.5 (39)	24.6	31.9
Maculopapular eruption	50.7 (35)	10.1	40.6
Chills	36.2 (25)	24.6	11.6
Red eye syndrome	33.3 (23)	33.3	0
Vertigo	29.0 (20)	20.3	8.7
Oedema (facial)	23.2 (16)	23.2	0
Vomiting	23.2 (16)	14.5	8.7
Babinski sign	20.3 (14)	20.3	0
Panic attack	18.8 (13)	14.5	4.3
Nausea	18.8 (13)	13.0	5.8
Hypotension	15.9 (11)	13.0	2.9
Hallucinations	10.1 (07)	8.7	1.4
Delirium	10.1 (07)	0	10.1
Nucal rigidity	10.1 (07)	10.1	0
Aggressive behaviour	07.2 (05)	4.3	2.9

**Table 4:** Anamnesis, physical examination, and laboratory variables obtained during admission and corresponding statistical tests for association with the development of ES or death from ES.

Variable	Encephalopathy			Death		
	**OR**	**95% CI**	***p***	**OR**	**95% CI**	***p***
**Anamnesis**						
*Oedema*	**4.0**	**1.0–15.4**	**0.03**	**1.8**	**0.2–11.5**	**0.09**
*Bone pain*	2.3	1.2–4.3	0.10			
*Paralysis*	3.0	0.4–22.0	0.20			
*Hypoestesia*	2.0	0.3–12.2	0.40			
*Abdominal pain*				**4.5**	**1.5–14.0**	**0.006**
*Conjunctivitis*				**3.0**	**1.0–8.7**	**0.03**
*Pruritus*				**3.0**	**1.0–8.7**	**0.04**
*Adenomegaly*				2.2	0.7–6.1	0.1
*Weight loss*				2	0.6–6.6	0.2
*Weakness/asthenia*				2	0.5–7.4	0.3
*Diarrhoea*				4.9	0.9–25.6	0.4
**Physical examination**						
*Apathy*	**1.9**	**1.0–3.8**	**0.04**			
*Depression*	**1.9**	**0.9–4.2**	**0.07**			
*Facial paralysis*	3.5	0.6–17.8	0.1			
*Incomprehensible Speech*	2.2	0.5–7.7	0.2			
*Babinski*	2.2	0.6–7.7	0.3	3.6	0.4–36.7	0.2
*Pale mucosa*				2.4	0.2–27.7	0.4
*Neck rigidity*				2.4	0.2–27.7	0.4
*Splenomegaly*				2.8	0.2–33.6	0.4
*Agitation*				2.4	0.2–27.7	0.5

**Table 5:** Frequency of deducted haplotypes in cases and controls, with OR and CI. (*) indicates Fisher’s test was used. LD: linkage disequilibrium.

	Cases	Controls	*p*	OR	CI	LD Cases
C*14/B*15	6/62	3/189	0.008 (*)	6.64	1.35–41.96	0.038
A*23/C*14	3/62	1/189	0.04 (*)	9.56	0.74–50.4	0.01
A*23/B*15	13/62	22/189	0.06	2.0	0.88–4.56	0.04
DR*07/B*58	5/62	5/189	0.07 (*)	3.23	0.71–14.49	0.017
